# Relative proportion and contrasting host preference of *Culex pipiens* biotypes across Europe

**DOI:** 10.1186/s13071-026-07375-4

**Published:** 2026-04-01

**Authors:** Rohan Menon, Stefanos S. Andreadis, Constantianus J. M. Koenraadt, Niels O. Verhulst, Rickard Ignell, Sharon R. Hill

**Affiliations:** 1 Unit of Chemical Ecology, Department of Plant Protection Biology, Swedish University of Agricultural Sciences, Alnarp, Sweden; 2 Max Planck Center next Generation Insect Chemical Ecology (nGICE), Alnarp, Sweden; 3 Hellenic Agricultural Organization-Dimitra, Institute of Plant Breeding and Genetic Resources, Thermi, Greece; 4 Laboratory of Entomology, Wageningen University and Research, Wageningen, The Netherlands; 5 National Centre for Vector Entomology, Institute of Parasitology, Vetsuisse and Medical Faculty, University of Zürich, Zurich, Switzerland; 6 One Health Institute, Vetsuisse Faculty, Faculty of Medicine and Faculty of Science, University of Zurich, Zurich, Switzerland

**Keywords:** Pipiens, Molestus, Host preference, Relative proportion, Human odor, Chicken odor

## Abstract

**Background:**

The primary vector of West Nile virus (WNV) in Europe, *Culex pipiens*, has two morphologically identical but behaviorally and genetically distinct biotypes, here referred to as Pipiens and Molestus. Pipiens and Molestus, and their hybrids, are differentially distributed across Europe and display variable patterns of blood-feeding on birds and humans across the continent, but whether host choice correlates with host preference is unclear.

**Methods:**

Samples of mosquitoes were collected and subsequently biotyped using real-time PCR, following which the relative proportions of each biotype and the hybrids was recorded and their host preference analyzed using a two-choice trapping assay. Each trapping assay consisted of two BG-Sentinel type 2 traps, which were baited with CO_2_ and either a synthetic human odor blend or a chicken odor blend. The trapping assays were conducted in peri-urban sites in the Netherlands, Switzerland and Greece.

**Results:**

The relative proportions of Molestus and hybrids were higher in Greece than in the northern locations, while Pipiens remained the dominant biotype across all trapping locations. In Greece, the host preference of Pipiens and Molestus was for avian and human odors, respectively, whereas the host preference was reversed in the Netherlands and Switzerland. The hybrids were opportunistic in host preference regardless of trapping location.

**Conclusions:**

The relative proportions of Pipiens and Molestus and their hybrids vary across Europe. The observed variance in host preference, ranging from opportunistic to weakly ornithophilic for Pipiens, from ornithophilic to mildly anthropophillic for Molestus and opportunistic for hybrids—depending on latitude—may have an impact on WNV transmission. This study highlights the discrepancy between host choice and host preference, and the efficacy of the synthetic host odor blends for surveilling the relative proportion and host preference of *Cx. pipiens.* This methodology provides a framework and the tools required for a more accurate assessment of vectorial capacity and prediction of WNV outbreaks, and may be used to understand the genetic mechanisms regulating host preference.

**Graphical Abstract:**

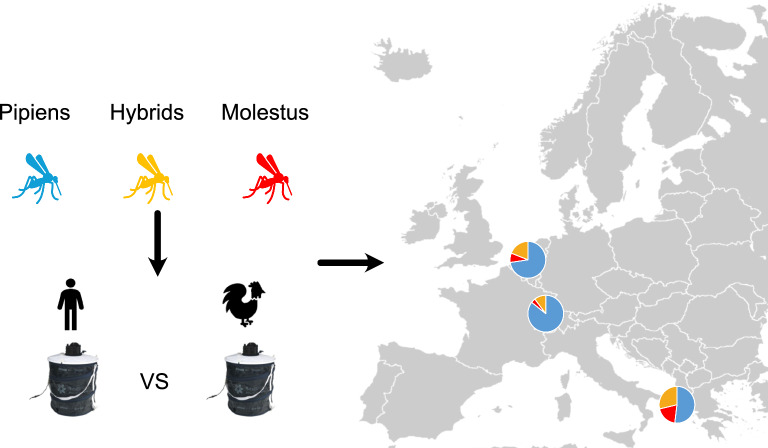

**Supplementary Information:**

The online version contains supplementary material available at 10.1186/s13071-026-07375-4.

## Background

**Fig. 1:**
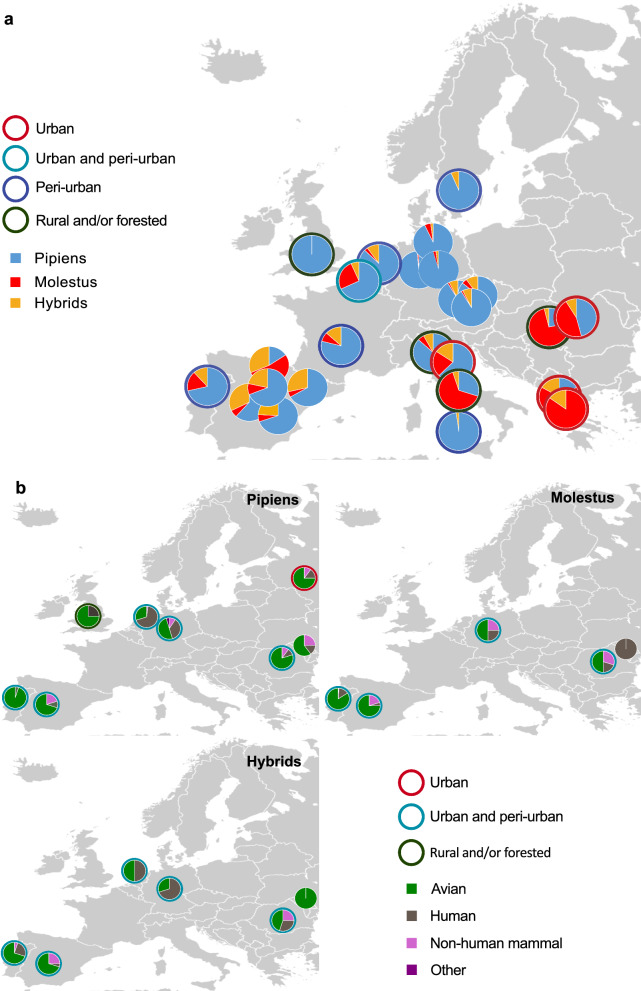
Relative proportion and blood-feeding patterns of the two *Culex pipiens* biotypes (Pipiens and Molestus) and their hybrids across Europe. **a** Relative proportion of Pipiens, Molestus and their hybrids across Europe based on data aggregated in Haba and McBride [3]. Colored rings around the pie charts indicate the ecology of trapping sites for each study. **b** Blood-meal analysis identifying the host choice of Pipiens (top left), Molestus (top right) and their hybrids (bottom left) from data aggregated in Wehmeyer et al. [7]. Colored rings around charts indicate the ecological context of the trapping sites, and colors within each pie chart represent identified host species

The primary vector of West Nile Virus (WNV) in Europe, *Culex pipiens*, has two morphologically identical but behaviorally and genetically distinct biotypes, referred to as *Cx. pipiens* f. *pipiens* (hereafter Pipiens) and *Cx. pipiens* f. *molestus* (hereafter Molestus) [1–3]. While both biotypes are competent vectors of WNV, their distribution differs across Europe (Fig. 1a) [3]. Pipiens and Molestus demonstrate differences in host preference under laboratory conditions [4–6], whereas the presumed avian-preferring (ornithophilic) Pipiens and human-preferring (anthropophillic) Molestus demonstrate a high variability in host choice across Europe (Fig. 1b) [3, 7]. While various factors, such as host availability, may explain the discrepancy between laboratory preference and field observations of choice, there is currently no experimental evidence for host preference of the biotypes under field conditions, and how this differs geographically. Using host choice as a proxy may restrict our understanding of how the differential host preference of the biotypes and their hybrids contributes to differential WNV transmission across Europe.

The distribution, habitat preference (Fig. 1a) and relative population size of Pipiens and Molestus vary across Europe [2, 3, 7]. Pipiens is distributed throughout Europe in multiple different habitats (Fig. 1a) [2, 7, 8]. In contrast, Molestus is more prevalent in south-eastern Europe, although in similar habitats as Pipiens (Fig. 1 a) [3], with smaller populations being detected in urban or peri-urban habitats in northern Europe within the last two decades [3, 7–10]. In northern Europe, the emergence of Molestus has been linked to increased travel and trade, leading to hybrids of Pipiens and Molestus where the two biotypes are sympatric [3, 7]. The role of these hybrids as bridge vectors for WNV has been theorized [11–14]. Therefore, an increased understanding of their host preference is required.

Pipiens, Molestus and their hybrids demonstrate differences in host choice, i.e. the ability to detect and feed on available hosts [15, 16], with blood-meal analyses of field-collected mosquitoes predominantly identifying birds, but also humans and non-human mammals, as hosts (Fig. 1b) [7]. For Pipiens and hybrids, host choice seems to differ geographically, with the incidence of human feeding being more prominent in north-western Europe, while no clear pattern of host choice is seen for Molestus (Fig. 1b) [7]. Compared to field observations using host choice, laboratory colonies of Pipiens and Molestus demonstrate different levels of preference for avian and human hosts, respectively, depending on the geographic origin of the populations [4–6]. Using host choice as a measure of host preference is problematic, as the host choice is influenced by numerous factors, including host availability [16], whereas host preference assesses the ability of mosquitoes to select a host over others when provided equal access [15].

The aim of this study was to assess the host preference in the context of the relative proportions of Pipiens, Molestus and their hybrids trapped across a latitudinal gradient in Europe. To overcome the intrinsic problem with host availability, we used a two-choice trapping system baited with synthetic human and chicken odors [6, 17, 18]. Specimens were collected over 12 days per country and subsequently biotyped. Next, their host preference was determined and compared across countries. Observed differences in relative distribution and host preference of the biotypes and hybrids of *Cx. pipiens* may be used to better understand the ongoing spread of WNV across Europe.

## Methods

### Field collection

To test if the host preference of the two biotypes of *Cx. pipiens* and their hybrids changes as latitudes increase across Europe, host-seeking *Cx. pipiens* were collected in Wageningen, the Netherlands (51.98° N, 5.66° E), in Zürich, Switzerland (47.38° N, 8.54° E) and in Thessaloniki, Greece (40.67° N, 22.79° E). Within a peri-urban area in each city, six trapping sites were selected, with each trapping site situated at least 100 m apart. Trapping sites were selected based on the availability of potential avian and human hosts, with traps placed under the cover of vegetation to prevent disturbance during trapping.

At each of the six trapping sites, two BG-Sentinel type 2 (Biogents AG, Regensburg, Germany) traps were set 1–2 m apart, at ground level. Both traps were connected to a single 10-l CO_2_ cylinder (flow rate 200 ml min^−1^, 99% purity) via Teflon™ tubing, and baited with one of two synthetic host odor blends or with a solvent control (heptane; 99% purity, Merck, Steinheim, Germany), which were released by diffusion from wick dispensers [19]. The synthetic human and chicken odor blends were prepared as previously described [17, 18] (Additional file 1: Table S1). The stock synthetic odor blends were diluted in heptane to obtain a release rate of 4 μl min^−1^ of the diluted blend. Wick dispensers were made from 4-ml glass vials with a cotton wick, encased in Teflon™ tubing, protruding through a pinhole in the cap. Dispensers were hung on the outside of the traps near the entrance, along with the tips of the tubes carrying the CO_2_.

Trapping was conducted for 12 days per country between the months of July and September 2023, following a Latin square design. In the Netherlands and Greece, treatment sites (i.e. a site containing two traps, one baited with the synthetic chicken odors and the second baited with synthetic human odor) and a control site (i.e. a site with traps only baited with CO_2_) were randomly assigned on the first day and then traps rotated over the following days between sites to minimize location bias. In Switzerland, control traps were run only on 1 day. Each day, traps were connected to a fully charged 12 V battery and set to operate from 2 h before sunset to 2 h after sunrise, after which they were shut down, and the captured mosquitoes collected. Collected mosquitoes were stored at - 20 °C and then morphologically identified under a microscope using the European identification key for female mosquitoes [20]. Only female *Cx. pipiens* mosquitoes were counted and subsequently stored at - 20 °C in 1.5-ml tubes for further molecular identification of biotypes and hybrids.

### Biotype identification

For the molecular analyses, samples were selected at random for the quantitative PCR (qPCR), with equal numbers of samples chosen from both odor-baited traps at each site and country. DNA was extracted using the Phire Tissue Direct PCR Kit (Thermo Fisher Scientific, Waltham, MA, USA). In short, two to three legs of each sampled mosquito were placed in a vial containing 20 μl of a mixture containing 0.5 μl of DNA Release and 19.5 μl dilution buffer. The vials were incubated at room temperature for 5 min and then placed in a thermocycler for 3 min at 95 °C to allow for DNA extraction. After extraction, DNA samples were stored at - 20 °C for future use.

Samples were biotyped as either Pipiens, Molestus or hybrids using a modified version of the real-time PCR assay described previously [8]. In short, universal forward and reverse primers Cx_pip_F (5′- CGGCCAAATATTGAGACTTTC-3′) and Cx_pip_R (5′-ACTCGTCCTCAAACATCCAGACATA-3′) were used for all samples. To identify biotype Molestus, probe Cpp_mol_P (5′- FAM-GAACCCTCCAGTAAGGTA-MGB-3′) was used; to identify Pipiens, probes Cpp_pip_P1 (5′-VIC-CACACAAAYCTTCACCGAA-MGB-3′) and Cpp_pip_P2 (5′-VIC-ACACAAACCTTCATCGAA-MGB-3′) were used. Samples that showed amplification for both Pipiens and Molestus probes were classified as hybrids. Samples were analyzed using a BioRad CFX96 real-time thermocycler (BioRad Laboratories, Hercules, CA, USA), at the following thermocycler conditions: 95 °C for 10 min, followed by 50 cycles of 95 °C for 15 s and 62 °C for 1 min. The data was analyzed using the BioRad CFX manager software (BioRad Laboratories). Samples with cycle threshold (Cq) values < 45, which showed exponential amplification, were treated as positive samples and used for further analysis. Negative controls and positive biotype controls, obtained from laboratory colonies of Pipiens and Molestus, were included in each PCR run.

### Statistics

Samples that were biotyped by qPCR were analyzed using a linear mixed model, followed by Tukey’s multiple comparisons post-hoc test, with R software (version 4.3.1) [21], using the ‘readxl,’ ‘lme4,’ ‘car,’ emmeans’ and ‘multicomp’ packages. The model included the interaction of location of trapping, lure odor and biotype identification (ID) of the samples. An analysis of variance (ANOVA) was subsequently run on the model to compare the variance of the samples in each location by biotype. To analyze the effect of the synthetic host odors on the number of collected *Cx. pipiens*, we created a linear model by an ANOVA, with Tukey’s post-hoc analysis. To analyze the host preference of the biotypes across countries, a preference index (PI) was calculated using the following formula PI = (H − C)/(H + C), in which H represents the number of mosquitoes of each biotype caught in the traps baited with synthetic human odor and C is the number of mosquitoes of each biotype caught in the traps baited with synthetic chicken odor. A linear model was created to compare the PI for each biotype across all locations using Tukey’s post-hoc analysis.

## Results

### Distribution of biotypes and hybrids across Europe

A total of 36,245 female *Cx. pipiens* were collected during 4320 trapping hours across the three European locations, with 24,161, 8297 and 3787 female *Cx. pipiens* mosquitoes collected at the sampling sites in Greece, Switzerland and the Netherlands, respectively (Additional file 2: Table S2). Of the total number of samples collected, 10% of those collected from Greece and 20% of those collected in Switzerland and the Netherlands, respectively, were used for biotype identification, to obtain similar sample sizes for statistical analysis. Samples that did not show clear amplification (< 1% of total samples tested per country) were excluded from further statistical analysis.

**Fig. 2:**
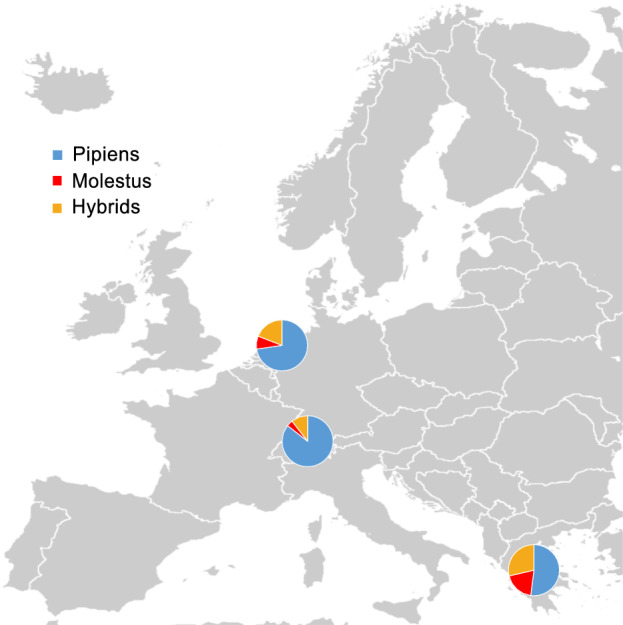
Relative proportion of *Culex pipiens* biotypes and hybrids captured in host odor-baited traps situated in three peri-urban locations across Europe. Pipiens, Molestus and hybrids were identified from a subset of collected samples from each country using quantitative PCR (*n* = 717, 1642 and 2196 mosquitoes for the Netherlands, Switzerland and Greece, respectively). Total number of collected Pipiens, Molestus and hybrids for each country are listed in Additional file 2: Table S2

Of the 4555 mosquitoes used for biotype identification across all three countries, 3067, 549 and 939 were identified as Pipiens, Molestus and hybrids, respectively (Fig. 2; Additional file 2: Table S2). Across all locations, Pipiens was the most abundant biotype, while Molestus was the least abundant (Additional file 2: Table S2). A linear mixed model demonstrated a significant difference in biotype and hybrid proportions across countries (*F*_(4, 60)_ = 97.6, *P* < 0.01), with no effect of trapping site on biotype distribution (*P* > 0.05). The proportion of Pipiens differed across the three countries (*χ*^2^ = 821.5, *df* = 2, *P* < 0.01), with Switzerland having the largest proportion of Pipiens, followed by the the Netherlands and then Greece with the lowest proportion (Fig. 2). In contrast, the proportion of Molestus and hybrids were higher in Greece than at the other two sampling locations, whereas the proportions did not differ between the Netherlands and Switzerland (*χ*^2^ = 226.7, *df* = 2, *P* < 0.01; *χ*^2^ = 241.2, *df* = 2, *P* < 0.01, respectively) (Fig. 2).

### Host preference of the biotypes and hybrids

**Fig. 3:**
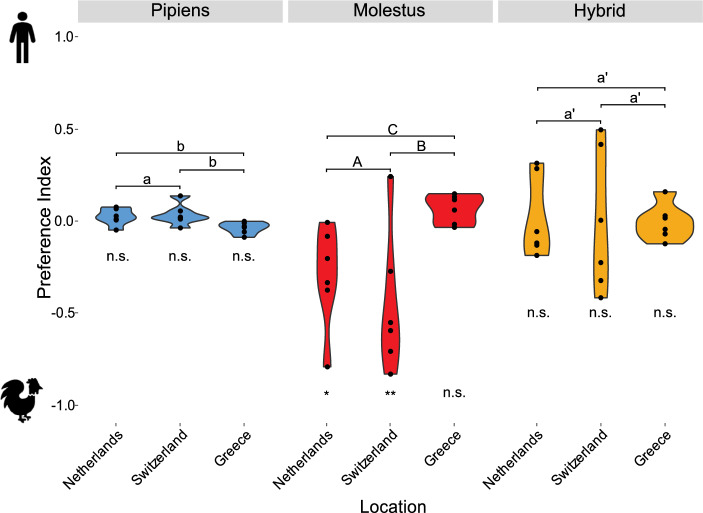
Host preference of *Culex pipiens* biotypes and hybrids across three locations in Europe, assessed using a two-choice trapping assay baited with synthetic chicken odor and human odor, respectively. Preference index ranges from − 1 (preference for chicken odor) to 1 (preference for human odor). Asterisks indicate the levels of significance for samples within each country (*P < 0.05 and **P < 0.01;* n.s.*, not significant), and letters within biotypes and hybrids above the graphs indicate the level of significance for comparisons between countries: a/A/a′ = not significant, b/B/b′ = significant at  *P* < 0.05, C = significant at  *P* < 0.01), calculated using a linear model with Tukey’s post-hoc test

The traps baited with the synthetic odor blends caught on average sixfold more mosquitoes than the CO_2_ controls (*F*_(1, 16)_ = 11.6, *P* < 0.01) (Additional file 2: Table S2), demonstrating the efficacy of the synthetic odors on trap catches. A general linear model on host preference identified a significant interaction between country and mosquito biotype/hybrid (*F*_(4, 30)_ = 3.2, *P* = 0.03); consequently, separate models were created for each biotype and the hybrids. While Pipiens showed no overall difference in host preference within and across the three locations (F_(2, 15)_ = 3.4, *P* = 0.06), a post-hoc analysis identified a marginally significant difference in the PI between the Netherlands and Greece (*P* ≤ 0.05) and a significant difference in the PI between Switzerland and Greece (*P* = 0.03), but not between the Netherlands and Switzerland (*P* = 0.78) (Fig. 3). The weak ornithophilic preference of Pipiens in Greece contrasts the weak anthropophillic behavior in the other two locations (Fig. 3). In contrast to Pipiens, the less numerous Molestus demonstrated an overall significant host preference across locations (F_(2, 15)_ = 5.3, *P* = 0.02), with a significant preference for the chicken odor-baited traps in the Netherlands (*P* = 0.02) and Switzerland (*P* < 0.01), but not in Greece (*P* = 0.60) (Additional file: Table S2). Moreover, there was a significant difference in the PI when the Netherlands and Greece were compared (*P* = 0.04) and when Switzerland and Greece were compared (*P* < 0.01), but not in the comparison of the Netherlands and Switzerland (*P* = 0.35). The weak anthropophillic preference of Molestus in Greece contrasts with the ornithophilic behavior in the other two locations (Fig. 3). As opposed to the two biotypes, the host preference of the hybrids did not differ within and across locations (*F*_(2, 15)_ = 0.01, *P* = 1.00) (Fig. 3).

## Discussion

The *Cx. pipiens* biotypes demonstrated similar relative proportion and host preference for human (Molestus) and chicken (Pipiens) odor in Greece, in line with previously reported studies on host choice at this latitude [3, 7, 22]. In contrast, in the two northern locations, the relative proportion of Molestus decreased, in line with previous reports across sites at these latitudes [3, 8, 10], while the host preference of both biotypes was inverted. Similar to Molestus, the relative proportion of hybrids decreased in the two northern locations, demonstrating an opportunistic host preference across these locations. These results support previous reports on the differential distribution of the biotypes and hybrids across Europe [3, 8], and provide novel information that is important for assessing the role of *Cx. pipiens* in WNV transmission dynamics.

The findings of this study show that the relative proportions of Pipiens, Molestus and hybrids vary significantly across Europe, with Pipiens being found throughout the continent in high numbers, and Molestus and the hybrids being more prevalent in south-eastern Europe (Fig. 1) [this study; 3, 8]. It remains unclear why Molestus has not spread in greater numbers throughout northern Europe [3]. Given the autogenous behavior of Molestus, its lack of a winter diapause and its ability to breed in more confined climate-regulated locations [23], this *Cx. pipiens* biotype should be well adapted to northern European habitats, although ecological [24], behavioral [1] or reproductive barriers [25, 26] may drive the separation of the two biotypes, even within populations in the same country [8, 27, 28]. Therefore, factors such as the ecological context of the trapping sites (e.g. degree of urbanization), type of traps used and the use of odor baits need to be considered, in order to ensure a more accurate representation of the relative population of host-seeking mosquitoes. Peri-urban sites were selected in the present study to represent areas with a likely presence of both biotypes and hybrids, as seen in prior studies [8, 22, 28]. The use of synthetic host odor blends, combined with CO_2_, increased trap capture, providing a stronger lure than CO_2_-baited traps [this study; 7, 18]. While the CO_2_ level used in this study was set to levels slightly lower than the typical CO_2_ emission levels of a human, and slightly higher than that of a chicken [29], of the hundreds of mosquitoes caught per trap pair, the host-odor baited traps successfully attracted on average more than sixfold more mosquitoes than the control. This leads to the conclusion that host-odor baited traps improve trap captures, which is important for accurate relative population surveys, providing data that can be used to assess the host preference, rather than the choice, of host-seeking mosquitoes.

The preference of Pipiens and Molestus for the chicken odor and human odor in Greece reflects previously reported results showing the preference of these biotypes for chicken and human odors in laboratory assays [5, 6], and in host choice studies in peri-urban locations from similar latitudes for Pipiens, but not for Molestus (Fig. 1b) [7, 22, 30, 31]. In the two northern locations, however, host preference of the two biotypes was juxtaposed with that observed in Greece, which corresponds to available data on host choice for Pipiens, for which sufficient data are available (Fig. 1b) [7, 10]. In contrast to the biotypes, the preference of the hybrids was opportunistic throughout the three locations, which is in line with available host-choice data (Fig. 1b) [7]. The selective pressures regulating the observed difference in host preference of the two biotypes and their hybrids across northern and southern latitudes are unknown, but have a genetic basis, as has been demonstrated for this and other mosquito species [32–34], and is likely driven by gene flow across populations. For the *Cx. pipiens* biotypes, laboratory assays demonstrate that differences in the strength of host preference depends on the origin of the populations, as well as the molecular correlates thereof [1–3]. Future work is required to assess the role of these and other molecular correlates in regulating the host preference of Pipiens and Molestus, with the aim to gain further insight into the mechanisms regulating WNV transmission by the biotypes and the hybrids across Europe.

The factors affecting the transmission of WNV by *Cx. pipiens* are complex [35], with prior research on the vector competence of *Cx. pipiens* highlighting Pipiens as the dominant WNV vector when compared to Molestus or the hybrids [14]. Previous studies have highlighted the effects of temperature and viral titer on vector competence [14] and on the factors regulating vectorial capacity, including the genetic origin of *Cx. pipiens* [36–38], host availability and feeding success on competent avian hosts [39]. Host preference [39], together with the relative abundance of the biotypes and hybrids and how these vary by geographical location, requires further analysis to create better models for understanding WNV transmission dynamics across Europe.

## Conclusions

The *Cx. pipiens* biotypes Pipiens and Molestus demonstrate significant variation in terms of relative proportion and contrasting host preference across Europe, while hybrids, though varying in relative proportion, display an opportunistic host preference regardless of trapping location. This variation in host preference may explain the varied host-feeding patterns of the biotypes and the hybrids reported in prior published studies. The efficacy of using synthetic host odors to assess host preference under field conditions provides tools for vector surveillance to assess relative population and host preference, with the aim of achieving better risk assessment of WNV transmission. In addition, the results of this study suggest that potential differences in the mechanisms regulating host preference depend on geographical location, which requires further analysis.

## Supplementary Information

**Additional file 1: Table S1. **Compounds used for the formulation of the synthetic human [17] and chicken [18] odor blends. Both odor blends used pentane as a solvent. All compounds were obtained from Merck, with purity ranging from 90% to 99%. Phenol and α-terpineol were obtained as solids, with the amount listed in micrograms.

**Additional file 2: Table S2. ***Culex pipiens *biotypes Pipiens and Molestus, as well as hybrids caught in the Netherlands, Switzerland and Greece. “Total captures” contains total numbers of mosquitoes caught in each country and the subset used for biotyping by qPCR are provided. “Trap catches” shows individual counts for each trap for all locations. “OdorvsControl” shows the total number of mosquitoes caught at each trapping site when used as a treatment (Odor) and as a control site for the Netherlands, Switzerland and Greece.

## Data Availability

All data generated or analyzed during this study are included in this published article [and its supplementary information files].

## Abbreviations

PI: Preference Index
WNV: West Nile virus
